# HIV burden and the global fast-track targets progress among pregnant women in Tanzania calls for intensified case finding: Analysis of 2020 antenatal clinics HIV sentinel site surveillance

**DOI:** 10.1371/journal.pone.0285962

**Published:** 2023-10-12

**Authors:** Erick Mboya, Mucho Mizinduko, Belinda Balandya, Jeremiah Mushi, Amon Sabasaba, Davis Elias Amani, Doreen Kamori, George Ruhago, Prosper Faustine, Werner Maokola, Veryeh Sambu, Mukome Nyamuhagata, Boniphace S. Jullu, Amir Juya, Joan Rugemalila, George Mgomella, Sarah Asiimwe, Andrea B. Pembe, Bruno Sunguya

**Affiliations:** 1 Muhimbili University of Health and Allied Sciences, Dar es Salaam, Tanzania; 2 Ministry of Health, Dodoma, Tanzania; 3 St. Francis University College of Health and Allied Sciences, Morogoro, Tanzania; 4 Tanzania Field Epidemiology and Laboratory Training Program, Dar es Salaam, Tanzania; 5 Muhimbili National Hospital, Dar es Salaam, Tanzania; 6 Centre for Diseases Control, Country Office, Dar es Salaam, Tanzania; 7 Global Fund, Tanzania Country Team, Tanzania; University of Zimbabwe Faculty of Medicine: University of Zimbabwe College of Health Sciences, ZIMBABWE

## Abstract

**Background:**

For successful HIV response, updated information on the burden and progress toward HIV elimination targets are required to guide programmatic interventions. We used data from the 2020 HIV sentinel surveillance to update on the burden and factors associated with HIV infection, HIV status awareness, and ART coverage among pregnant women in Tanzania mainland.

**Methodology:**

We conducted the surveillance in 159 antenatal clinics (ANC) from all 26 regions of Tanzania’s mainland from September to December 2020. This cross-sectional study included all pregnant women (≥15 years) on their first ANC visit in the current pregnancy during the survey period. Routine HIV counselling and testing were done at the facility. A multivariable logistic regression model accounting for the survey design was used to examine factors associated with HIV infections.

**Results:**

38,783 pregnant women were enrolled (median age (IQR) = 25 (21–30) years). HIV prevalence was 5.9% (95%CI: 5.3% - 6.6%), ranging from 1.9% in the Manyara region to 16.4% in the Njombe region. Older age, lower and no education, not being in a marital union, and living in urban or semi-urban areas were associated with higher odds of HIV infection. HIV status awareness among women who tested positive was 70.9% (95% CI: 67.5%- 74.0%). ART coverage among those aware of their status was 91.6% (86.5%- 94.9%). Overall, 66.6% (95% CI: 62.4%- 70.6%) of all pregnant women who tested positive for HIV knew their HIV status and were on ART.

**Conclusion:**

HIV is increasingly prevalent among pregnant women in Tanzania mainland especially among older, those with lower or no formal education, those outside marital union, and pregnant women living in urban and semi-urban areas. Behind the global fast-target to end HIV/ AIDS, about a third of pregnant women living with HIV initiating ANC were not on ART. Interventions to increase HIV testing and linkage to care among women of reproductive age should be intensified.

## Introduction

About 19.7 million women above 15 years were living with HIV in 2021, and about 1.3 million women become pregnant each year [1, 2]. The risk of transmission of HIV from mother to child ranges from 15% to 45% during pregnancy, labor, delivery, and breastfeeding if proper preventive measures are not taken [3, 4]. In 2020 alone, about 160,000 children had newly acquired HIV principally through vertical transmission, and about 1.8 million children were living with HIV worldwide [1]. The African region is the most affected region with 25.6 million people living with HIV (PLHIV) and about 60% of the global new HIV infections [2]. Despite the overall burden of HIV infection, women of reproductive age experience a higher burden as compared to men. In Africa, the prevalence of HIV among women of reproductive age ranges from <0.1% to 23.5% [5].

Tanzania is ranked among the 22 priority countries constituting 90% of all pregnant women living with HIV worldwide [6]. The country is ranked third among countries with the highest annual incidences of HIV globally. It recorded 72,000 new cases in the year 2018. Adolescents and young women contributed a disproportionately higher burden compared to other sub-populations [7]. About 36,700 women aged 15–49 were newly infected with HIV in 2020 and an estimated 28,000 in 2021 [5, 8]. Although the country has observed a significant reduction in mother-to-child transmission (MTCT) of HIV from 23% in 2011 to 11% in 2020, it is still behind the global targets and is one of the six countries with a slow pace or no decline in MTCT of HIV [6, 9].

Tanzania also focuses on ending HIV/AIDS as a public health threat by 2030. Reaching the fast-track targets calls for a better understanding of the local epidemic. The 95-95-95 fast-track targets aimed to ensure that 95% of PLHIV should know their HIV-positive status, 95% of people who know their status should be on treatment, and 95% of those on ART should attain and maintain viral suppression by 2030 [10]. Among adults living with HIV in 2016 in Tanzania, only 51.8% were aware that they were HIV-infected and 47.1% were aware and on ART [11]. Although women living with HIV were more likely to know their HIV-positive status and be on ART compared with the general population, the level of HIV status awareness and ART coverage was still very low. Only 55.4% of them were aware of their HIV-positive status and slightly over half (51.5%) of those infected were on ART [11]. This hampers efforts to eliminate HIV/AIDS by 2030 [12, 13].

For over two decades, the HIV sentinel surveillance (HSS) among pregnant women attending ANC has provided estimates of the burden and trend of HIV infections in Tanzania [14–17]. Six rounds of HSS have been done since 2000 and results indicate a decline in the prevalence of HIV infections among pregnant women in Tanzania from 9.6% in 2001 to 5.6% in 2011 [16]. However, results from the last HSS, in 2017 (unpublished), showed an increase in prevalence to 6.1%. The HSS also plays a vital role in informing the need for HIV testing, and other HIV and STIs-related needs at the national and sub-national levels to prevent mother-to-child transmission (PMTCT) of HIV [14, 18]. The HSS has also been expanding to include all regions, representing facilities from urban, semi-urban, and rural areas. In line with the ambitious global targets, recent surveys also collected information on HIV status awareness and ART coverage among pregnant women which help to inform on the progress towards the goals and effectiveness of the interventions.

In line with the need to close the gap in HIV control, essential to the national and global response to HIV, we analyzed data from the seventh HSS round conducted in 2020 to update national data on the burden and factors associated with HIV infection, awareness of HIV status, and coverage of ART among pregnant women in Tanzania mainland.

## Materials and methods

### Surveillance duration and site selection

The seventh HSS was conducted from September to December 2020 in 26 regions of Tanzania’s mainland from 159 antenatal clinics (ANCs) providing PMTCT services. As for the previous HSS rounds, two urban, two semi-urban, and two rural sites were conveniently selected from each region, except for the Dar es Salaam region, where all six sites are classified as urban. ANCs in the seventh HSS round included 148 ANCs that participated in the sixth HSS (conducted in 2017). Ten ANCs were purposively selected to replace 10 ANCs that participated in 2017 and were no longer providing ANC services during the current survey period. One urban ANC was newly added to the Geita region which had only one urban ANC site in the previous round of HSS.

ANC coverage of at least one visit is more than 98% and PMTCT services are integrated into ANCs [9]. In 2019, 2.2 million pregnant women accepted HIV testing, and 98.3% of all pregnant women attended PMTCT services [9]. Pregnant women with HIV-positive test results initiate ART irrespective of their CD4 cell count, in line with the national guidelines [19]. Women who opt out at their first ANC visit are informed that they can access HIV testing at any future visit if they change their minds [19].

### Survey population

All pregnant women aged 15 years and above attending ANC on their first visit in the current pregnancy during the survey period were eligible for inclusion in the ANC HSS. However, only those who provided written consent and were willing to give a blood sample for routine PMTCT HIV testing were included in the surveillance. To achieve the targeted sample size, all sites carried out data collection continuously for three consecutive months. Assuming an alpha level of 0.05, with 159 ANCs sampled, and 38,755 women included in the analysis with an intracluster correlation of 0.16, this survey had the power of ≥80% to detect at least a 2.5% change in the prevalence of HIV as that of 6.3% reported in 2017.

### Data collection

This surveillance was integrated into routine ANC activities where all women attending the ANC on a particular day were informed of the ongoing surveillance. The provider explained the surveillance procedures and obtained written informed consent from the participant for each component of the survey (the interview and routine rapid testing for HIV). In addition, a non-identifying unique survey ID code (HSS number) pre-printed on barcode stickers was assigned to each prospective survey participant found to be eligible.

The surveillance questionnaire was developed in English, translated into Swahili, and then back-translated into English by a third party to verify the accuracy of the translation. A trained RCH provider, using an electronic tablet loaded with an open data kit (ODK), a data collection software, administered the questionnaire. It captured the HSS number, ANC number, age, marital status, gravidity, education level, residence, employment, HIV status, ART use, and HIV test results. Electronic data were uploaded to a password-protected server at the end of each day. Details about the training of the study personnel have been described elsewhere [20].

### HIV testing procedures

Following the interview, all participants, including those who knew their HIV status, were offered HIV counselling and testing according to the national guidelines and the national rapid testing algorithm [21]. The provider conducted HIV and syphilis testing using rapid SD Bioline HIV/ Syphilis Duo test kits, followed by a Unigold test to confirm any HIV-positive results. The SD Bioline HIV/ Syphilis Duo test kit offers similar sensitivity and specificity for HIV as the single HIV SD Bioline [22–25]. This allows for comparability with the results of the previous rounds of this surveillance. The Ministry of Health (MOH) adopted the kit owing to its cost-effectiveness in testing for both HIV and syphilis and enables broader syphilis testing coverage [23, 24]. Each participant received their result in a post-test counselling session and was referred to HIV prevention, care, treatment, and support services as appropriate. Participants with positive syphilis test results were treated according to National STI Management Guidelines [26]. HIV test result data were recorded on a register and entered into an ODK form.

### Data management and analysis

Sociodemographic data and routine HIV testing data collected were uploaded daily to a password-protected cloud storage system by each survey team member to allow for real-time data monitoring. To minimize data entry errors, questions in the ODK had prompts and checks for data validation and correction of identified errors. Data registries at the facility and monthly reports submitted to the MoH were also referred to ensure the quality of the study data entered.

Analysis was conducted using Stata Statistical software ver. 17 (College Station, TX: Stata Corp LLC.). The proportion of women who were infected with HIV was calculated at the national and regional levels and was also disaggregated by demographic characteristics. These estimates, and the logistic regression estimates, considered the survey design of this surveillance (clustering at the health facility level and stratification by locality—rural, semi-urban, and urban). The estimates were also weighted using inverse probability weights based on the total number of women who attended ANC during the study period at each region. Weights did not take into account non-response since it was so low (<2%). To compare with previous surveys, crude estimates (unweighted and not considering the survey design) of HIV infection at the national level were calculated and presented. Multivariable logistic regressions were used to examine the independent association between HIV infection and various independent variables including age, marital status, parity, education level, source of income, and locality. All the variables that attained a p-value <0.2 in the univariable analysis were included in the multivariable models. The level of statistical significance was set at p<0.05.

We also estimated the proportion of HIV-positive status awareness among women living with HIV in the survey and the ART coverage among those aware of their HIV-positive status. These estimates were disaggregated by regions and by demographic characteristics of the women. Because the HIV status awareness and ART coverage were self-reported, and no efforts were made to verify the responses, we conducted and presented the sensitivity analysis of their estimates. For ART coverage, we estimated and presented estimates where 10% of those who reported being aware of their status but not on ART were considered to be on ART, and 5% of those who reported being aware of their HIV status and on ART were considered to be not on ART [11]. For HIV-positive status awareness, 10% of those who reported being unaware of their HIV-positive status were considered to have previously been diagnosed. These estimates were derived from the 2016/17 Tanzania HIV Impact survey [11].

### Ethical consideration

Study staff sought informed consent from potential participants before they engaged them in the survey. Pregnant women who did not consent to participate in the survey received routine ANC care as expected. Participants were assured that their responses would be kept confidential and that no harm would come to them because of agreeing or not agreeing to participate in the survey. Approval to conduct this study was provided by the MUHAS Institutional Review Board (MUHAS-REC-07-2020-298). Strict protection ensured data security and confidentiality. The ANC HSS survey did not collect any personally identifiable information other than what was essential to meet the assessment objectives.

## Results

### Demographic characteristics

Of the 39,516 pregnant women included in the 2020 ANC HSS surveillance, 38,783 (98.1%) consented to participate in the surveillance and had their HIV results. The median age (IQR) of the participants was 25 (21–30) years, and a third of them (33.2%) were aged between 20–24 years. Over three-quarters of the participants (78.5%) reported being married, and 61.0% had attained primary school education. About half of the participants (48.8%) were housewives, while 44.5% had at most two previous pregnancies, and a quarter of all participants (26.3%) were primigravids. Almost half of the participants, 48.2%, were recruited from facilities located in urban areas—**Table 1**.

**Table 1:** Prevalence of HIV among pregnant women initiating ANC by their demographic characteristics (N = 38783).

Variable	Total	HIV Infection
Age group (years)	N (%)	Prevalence (%) (95% CI)
15–24	17,766 (45.8)	3.0 (2.6–3.4)
25–34	15917 (41.0)	7.4 (6.6–8.2)
35–44	4746 (12.2)	11.2 (9.9–12.7)
45+	354 (0.9)	18.2 (12.1–26.4)
**Education level**		
None	4,896 (12.6)	7.2 (6.2–8.4)
Primary	23,627 (60.9)	6.8 (6.1–7.5)
Secondary	9,035 (23.3)	4.9 (4.1–5.9)
Post-secondary	1,225 (3.2)	4.7 (3.5–6.1)
**Marital status**		
Single	2,835 (7.3)	10.4 (8.7–12.4)
Divorced/separated	308 (0.8)	22.4 (16.3–29.9)
Widowed	113 (0.3)	23.0 (16.5–31.2)
Co-habiting	5,081 (13.1)	6.8 (5.9–8)
Married	30,446 (78.5)	5.6 (5.1–6.3)
**Employment status**		
Employed	2,014 (5.2)	7.7 (6.2–9.5)
Housewife	18,956 (48.9)	5.4 (4.9–6)
Self-employed	15,032 (38.8)	7.5 (6.6–8.5)
Unemployed	2,781 (7.2)	5 (3.7–6.7)
**Total number of previous pregnancies**		
0	10,199 (26.3)	2.7 (2.3–3.1)
1–2	17,285 (44.6)	7.1 (6.4–7.8)
3–4	7,916 (20.4)	9.6 (8.6–10.8)
>4	3,383 (8.7)	6.0 (5.1–7.0)
**Locality**		
Rural	6,925 (17.9)	4.3 (3.4–5.3)
Semi-urban	13,020 (33.6)	7.3 (6–8.9)
Urban	18,838 (48.6)	6.4 (5.6–7.3)
**Health facility level**		
Dispensary	6,131 (15.8)	5.5 (4.3–6.9)
Health Centre	23,837 (61.5)	5.9 (5.2–6.8)
Hospital	8,815 (22.7)	8.0 (6.7–9.7)

ANC- Antenatal clinic, CI- Confidence Interval

### Prevalence of HIV by demographic characteristics of participants

The overall prevalence of HIV infection among first-visit ANC attendees in Tanzania mainland was 5.9% (95%CI: 5.3% - 6.6%). The prevalence of HIV was highest among women aged 45 years and above (18.2%), widowed women (23.0%), and divorced/separated women (22.4%). Women in semi-urban areas were observed to have a higher prevalence (7.3%) compared to those in urban (6.4%) and rural areas (4.3%). Women in their first pregnancy had the lowest prevalence compared to women with previous pregnancies—**Table 1**. The crude prevalence of HIV was 6.3% (95%CI: 6.0%- 6.5%).

### Prevalence of HIV infection by regions

The prevalence of HIV infection was unevenly distributed ranging from the lowest in the Manyara region (2.0%) to the highest in the Njombe region (16.4%)—**Table 2.**

**Table 2:** Prevalence of HIV infection among pregnant women initiating ANC in Tanzania mainland by regions (N = 38783).

Region	Total N	HIV Infection	
		n	%, (95% CI)
Arusha	1,542	51	3.3 (1.7–6.4)
Dar es Salaam	2,874	167	5.8 (4.3–7.9)
Dodoma	1,749	68	3.9 (3.5–4.3)
Geita	3,460	231	6.7 (5.6–8)
Iringa	882	120	13.6 (10.9–16.9)
Kagera	1,481	73	4.9 (3.2–7.5)
Katavi	1,224	72	5.9 (4.3–8.1)
Kigoma	1,372	29	2.1 (1.1–4.1)
Kilimanjaro	968	68	7 (4.3–11.2)
Lindi	508	36	7.1 (5.7–8.8)
Manyara	924	18	1.9 (1.0–3.6)
Mara	1,509	83	5.5 (2.8–10.5)
Mbeya	2,032	241	11.9 (9.7–14.4)
Morogoro	728	35	4.8 (2.2–10.4)
Mtwara	814	39	4.8 (3.9–5.9)
Mwanza	2,232	128	5.7 (4–8.1)
Njombe	670	110	16.4 (11.2–23.5)
Pwani	1,841	149	8.1 (3.8–16.5)
Rukwa	1,265	80	6.3 (4.6–8.7)
Ruvuma	1,119	105	9.4 (4.6–18.2)
Shinyanga	1,477	102	6.9 (3.9–11.8)
Simiyu	1,956	85	4.3 (2.7–6.8)
Singida	1,549	67	4.3 (2.4–7.8)
Songwe	1,902	132	6.9 (5.3–9.1)
Tabora	1,408	96	6.8 (4.6–10.1)
Tanga	1,297	71	5.5 (4.4–6.8)
**Total**	**38,783**	**2456**	**5.9 (5.3–6.6)**

ANC- Antenatal Clinic, CI- Confidence interval

### Factors associated with HIV infection among pregnant women

**Table 3** shows the results of logistic regression analyses. The odds of being HIV positive increased with age (trend z = 26.02, p<0.001). Pregnant women aged 25–34 years had 2.2 times higher odds of having HIV than pregnant women aged 15–24 years (aOR = 2.2, 95% CI: 1.9–2.5). Similarly, women aged 35–44 years (aOR = 3.9, 95% CI: 3.2–4.6), and women of 45 years and above (aOR = 7.0, 95% CI: 4.4–11.1) had respectively almost 4 and 7 times higher odds of having HIV infection compared to pregnant women aged 15–19 years (**Table 3**). The odds decreased with an increase in education level (trend z = -4.54, p<0.001).

**Table 3:** Factors associated with HIV infection among pregnant women initiating ANC in Tanzania mainland, (N = 38783).

Variable	Positive n (%)	cOR (95% CI)	aOR (95% CI)	p-value
**Age group (years)**				
15–24	582 (3.3)	Ref	Ref	
25–34	1246 (7.8)	2.6 (2.3–2.8)	2.2 (1.9–2.5)	<0.001
35–44	557 (11.7)	4.1 (3.5–4.7)	3.9 (3.2–4.6)	<0.001
45+	71 (20.1)	7.2 (4.4–11.7)	7.0 (4.4–11.1)	<0.001
**Education level**				
None	353 (7.2)	1.6 (1.2–2.2)	2.3 (1.6–3.4)	<0.001
Primary	1599 (6.8)	1.5 (1.1–2)	1.9 (1.3–2.6)	<0.001
Secondary	447 (5.0)	1.1 (0.8–1.5)	1.2 (0.9–1.7)	0.227
Post-secondary	57 (4.7)	Ref	Ref	
**Marital status**				
Single	294 (10.4)	1.9 (1.6–2.3)	3.3 (2.7–4.0)	<0.001
Co-habiting	348 (6.9)	1.2 (1–1.5)	1.4 (1.2–1.7)	0.001
Divorced/separated	69 (22.4)	4.8 (3.2–7.2)	4.8 (3.2–7.1)	<0.001
Widowed	26 (23.0)	5 (3.2–7.7)	3.4 (2.2–5.4)	<0.001
Married	1719 (5.7)	Ref	Ref	
**Employment status**				
Employed	155 (7.7)	1.6 (1.2–2.2)	1.7 (1.1–2.4)	0.008
Housewife	1032 (5.4)	1.1 (0.8–1.5)	1.2 (0.9–1.7)	0.262
Self-employed	1131 (7.5)	1.6 (1.1–2.1)	1.6 (1.1–2.2)	0.012
Unemployed	138 (5.0)	Ref	Ref	
**Total number of previous pregnancies**				
0	271 (2.7)	Ref	Ref	
1–2	1219 (7.1)	2.8 (2.4–3.2)	2.2 (1.9–2.6)	<0.001
3–4	763 (9.6)	3.9 (3.3–4.6)	2.2 (1.8–2.6)	<0.001
>4	203 (6.0)	2.3 (1.9–2.9)	1.1 (0.8–1.4)	0.479
**Locality**				
Rural	296 (4.3)	Ref	Ref	
Semi-urban	954 (7.3)	1.8 (1.3–2.4)	1.5 (1.0–2.2)	0.030
Urban	1206 (6.4)	1.5 (1.1–2.1)	1.5 (1.1–2.1)	0.012
**Health facility level**				
Dispensary	335 (5.5)	Ref	Ref	
Health Centre	1412 (5.9)	1.1 (0.8–1.5)	1.1 (0.8–1.4)	0.669
Hospital	709 (8.0)	1.5 (1.1–2.1)	1.4 (0.9–2.0)	0.099

ANC- Antenatal clinic, cOR- Crude Odds Ratio, aOR- Adjusted Odds Ratio, CI- Confidence interval

Marital status was also a significant predictor of HIV infection among women in this survey. Widowed women were 3.4 times more likely to test HIV positive compared to those who were married (aOR = 3.4, 95%CI: 2.2–5.4). Similar findings were observed for those who were divorced/separated (aOR = 4.8, 95%CI): 3.2–7.1). Single women were three times more likely to test positive for HIV compared to married women (aOR = 3.3, 95%CI: 2.7–4.0). Compared to women in rural areas, women residing in urban and suburban areas had 50% higher odds of HIV infection **Table 3**.

### Awareness of HIV-positive status and ART coverage

**Fig 1:**
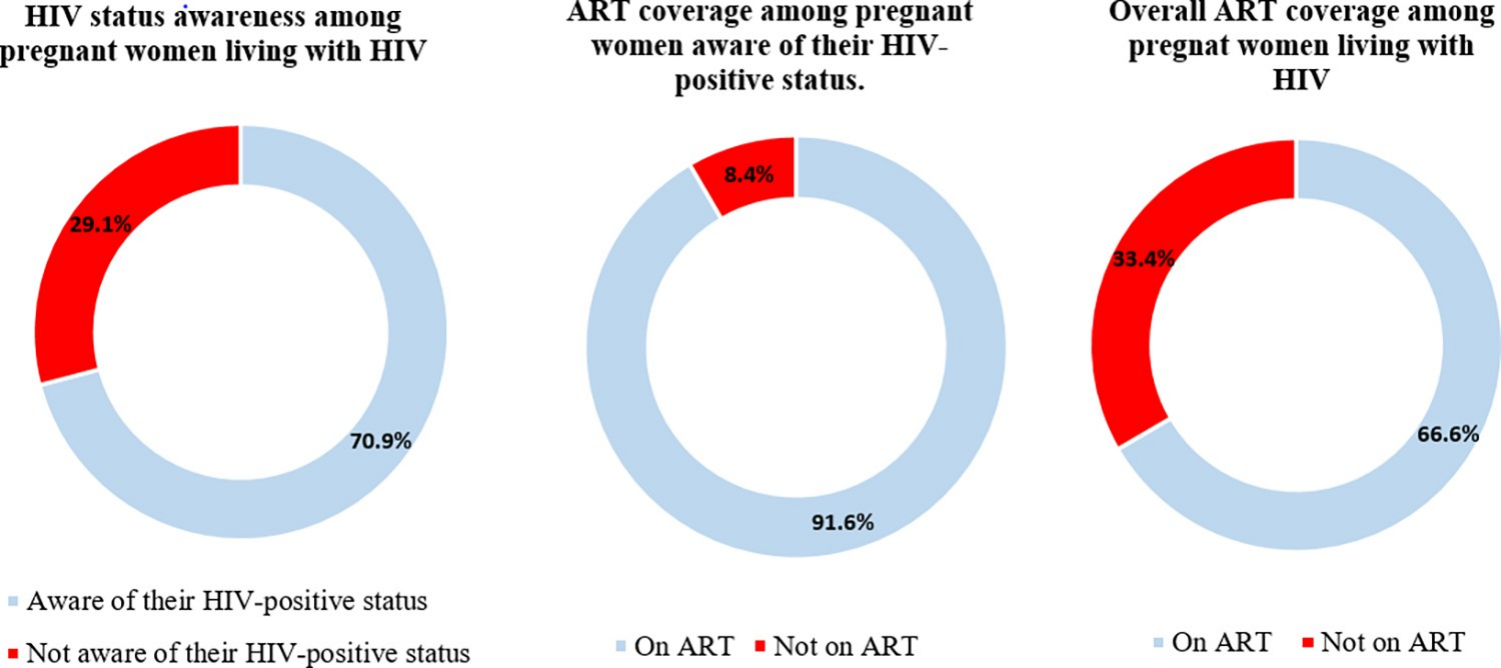
Progress on the global fast-track targets among pregnant women attending ANC in Tanzania’s mainland.

HIV status awareness among pregnant women who tested positive was 70.9% (95% CI: 67.5%- 74.0%). ART coverage among pregnant women aware of their status was 91.6% (86.5%- 94.9%). Overall, 66.6% (95% CI: 62.4%- 70.6%) of all pregnant women who tested positive for HIV in this survey knew their HIV status and were on ART—**Fig 1**.

In the sensitivity analysis of HIV status awareness, the HIV status awareness was 73.7% when 10% of HIV-positive women who reported being unaware of their HIV-positive status were considered to be aware of their HIV-positive status. And ART coverage was 87.1% in the sensitivity analysis when 10% of those who reported being aware of their HIV-positive status but not on ART were considered to be on ART, and 5% of those who reported being aware of their HIV status and on ART were considered to be not on ART.

### Awareness of HIV Positive Status and ART coverage by regions

**Fig 2:**
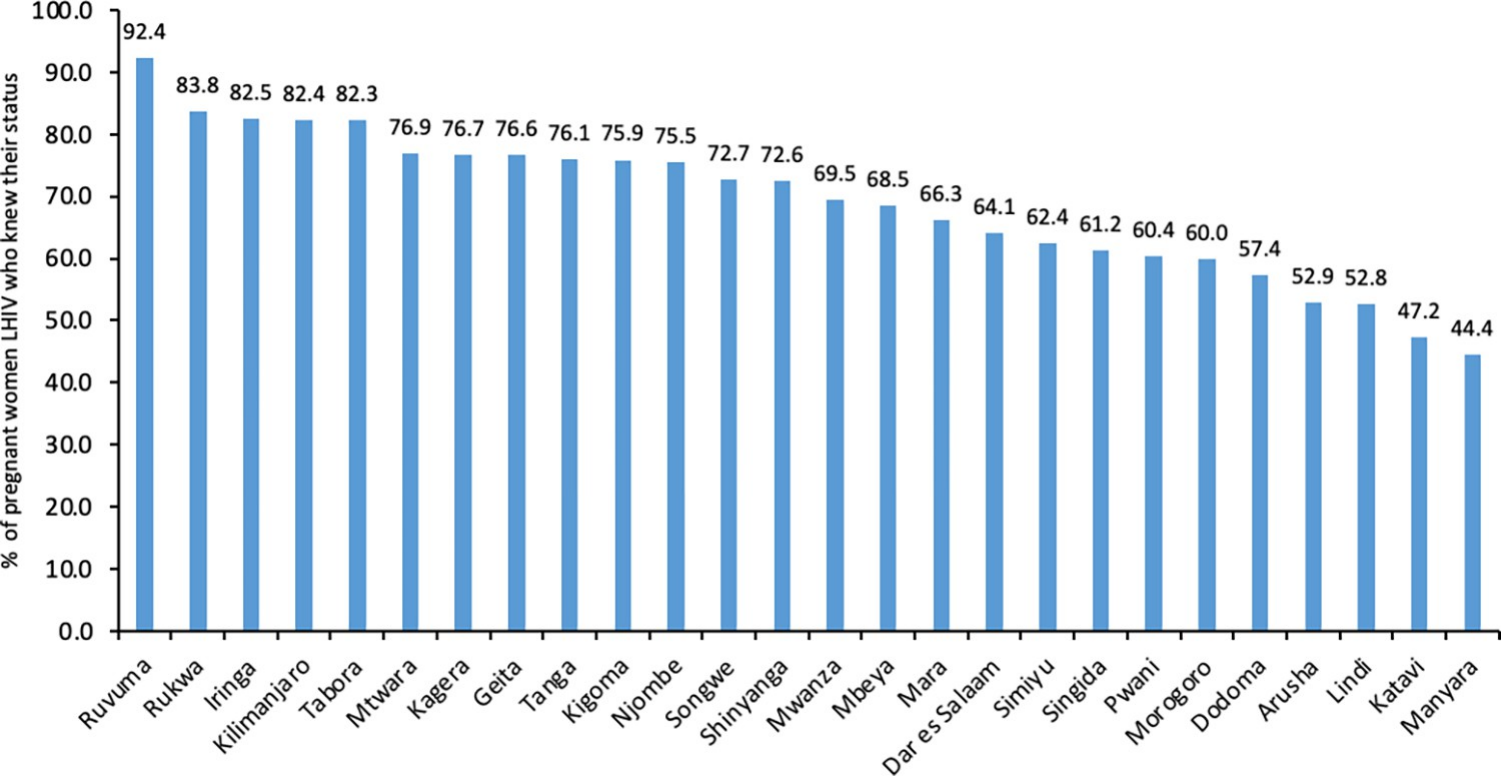
Percentage of HIV-positive women attending ANC who knew their status by region.

None of the regions has attained the first 95% target. The proportion of HIV-positive pregnant women attending ANC who knew their status varied among regions ranging from 92% in Ruvuma to 44% in Manyara and 47% in Katavi—**Fig 2**.

**Fig 3:**
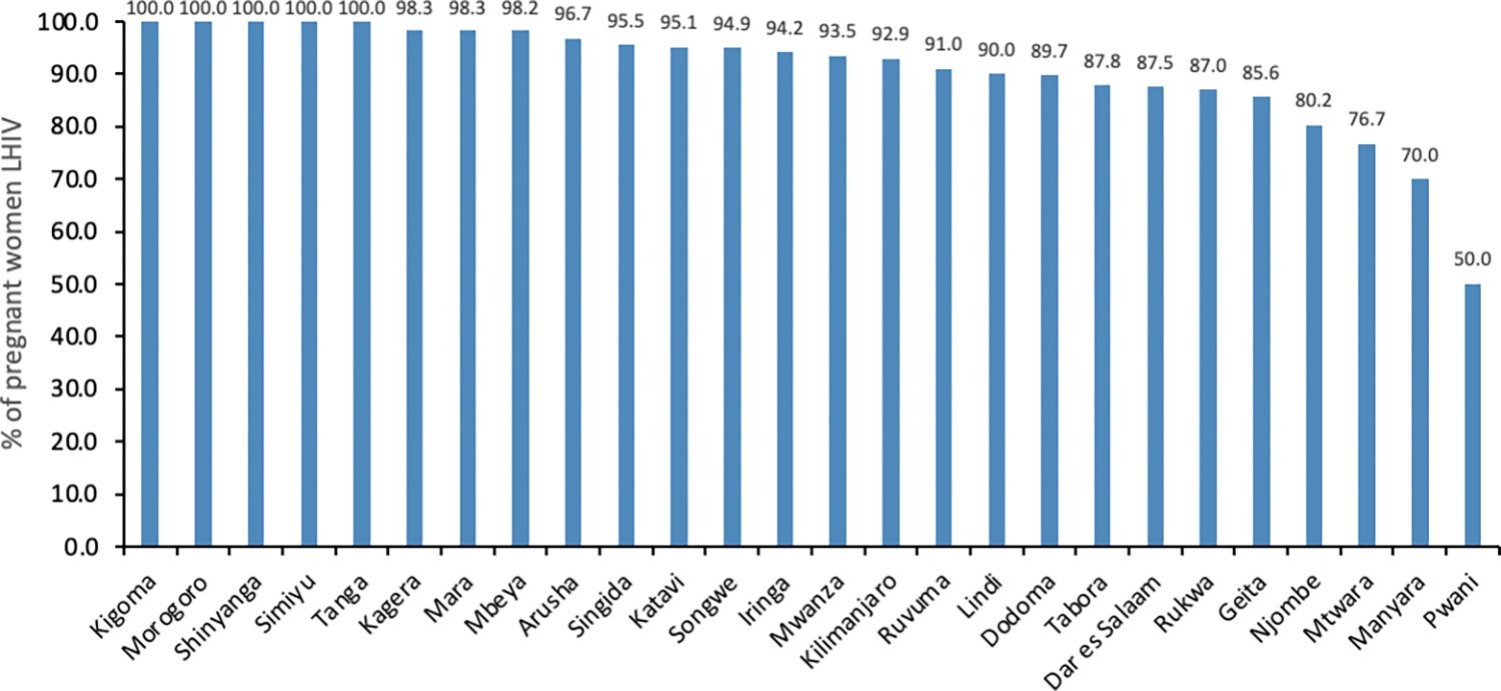
Percentage of HIV-positive women attending ANC who knew their status that were on ART by region.

Eleven regions had already attained the second 95% target. These were Kigoma, Morogoro, Shinyanga, Simiyu, Tanga, Kagera, Mara, Mbeya, Arusha, Singida, and Katavi. Three regions, Pwani (51%), Manyara (75%), and Mtwara (77%), had the lowest ART coverage. Dar Es Salaam (87%), Geita (86%), Lindi (89%), Njombe (83%), and Rukwa (87%) had ART coverage below 90% among women living with HIV who knew

their status—**Fig 3**.

### HIV Status awareness and ART coverage by demographic characteristics

Among pregnant women who tested positive for HIV, the HIV-positive status awareness was lowest (56.6%) among women aged 15–24 years, while the highest (80.4%) was among women aged 35–44 years. Less than half (46.6%) of primigravids who tested positive were aware of their HIV-positive status while over 70% of women with at least one previous pregnancy were aware of their HIV-positive status. Three-quarters (75.5%) of the pregnant women in semi-urban areas were aware of their HIV-positive status whereas, in comparison, two-thirds of those residing in the rural (66.4%) and urban areas (68.2%) were aware of their HIV-positive status.

ART coverage was highest among rural (97.1) residents and lowest among semi-urban dwellers (88.1%). ART coverage was lowest (30%) among pregnant women of 45 years and above. Women with secondary (85.5%) or post-secondary education (86.0%) had ART coverage lower than 90%. Primigravids had the lowest ART coverage (80.0%) compared to women with at least one previous pregnancy who had over 90% coverage—**Table 4**.

**Table 4:** HIV Status awareness and ART coverage by demographics.

Variable	Total Positive	Aware Status % (95% CI) N = 2456	On ART % (95% CI) N = 1708
**Age group (years)**			
15–24	582	56.6 (51.1–62.0)	90.9 (85.9–94.3)
25–34	1246	73.1 (69.1–76.7)	92.9 (89.3–95.4)
35–44	557	80.4 (75.7–84.3)	94.3 (88.9–97.1)
45+	71	68.8 (59.5–76.8)	30.3 (05.3–77.0)
**Education level**			
None	353	69.7 (63.4–75.4)	92.8 (86.2–96.4)
Primary	1599	72.1 (68.6–75.4)	93.1 (89.7–95.5)
Secondary	447	69.1 (63.2–74.5)	85.5 (71.2–93.3)
Post-secondary	57	55.6 (37.5–72.3)	86.0 (68.1–94.6)
**Marital status**			
Single	294	72.7 (59.0–83.1)	91.0 (62.5–98.4)
Co-habiting	348	64.9 (58.3–71.1)	94.9 (89.7–97.5)
Married	1719	72.1 (68.4–75.5)	91.0 (84.1–95.0)
Divorced/separated	69	72.3 (64.9–78.6)	91.7 (84.2–95.8)
Widowed	26	57.3 (33.5–78.1)	90.9 (71.0–97.6)
**Total number of previous pregnancies**			
0	271	46.6 (39.7–53.6)	80.0 (66.6–88.9)
1–2	1219	70.0 (66.0–73.6)	90.2 (84.1–94.1)
3–4	763	78.5 (74.6–81.9)	95.6 (91.7–97.8)
>4	203	81.6 (74.7–86.9)	93.9 (87.3–97.1)
**Locality**			
Rural	296	66.4 (59.9–72.3)	97.1 (91.9–99.0)
Semi-urban	954	75.5 (70.0–80.4)	88.1 (76.2–94.5)
Urban	1206	68.2 (63.3–72.8)	93.4 (89.3–96.0)

ART- Anti-retroviral therapy, CI- Confidence interval

## Discussion

HIV infection was prevalent in 5.9% of pregnant women attending ANC in Tanzania with a marked regional variation. This study further revealed that Tanzania is still far behind in attaining the first fast-track targets among pregnant women. HIV status awareness among pregnant women living with HIV in this survey was only at 70.9%, also with a wide regional variability. Among pregnant women who knew their HIV status, 91.6% were on ART. Age, marital status, education level, number of pregnancies, and locality were significant predictors of HIV infection among pregnant.

To compare the prevalence of HIV infection among pregnant women across rounds of HSS, we used the crude estimate. The crude prevalence in this survey was 6.3%, which is higher compared with the prevalence of the preceding survey of 6.1% reported in 2017 (unpublished). The trend of HIV prevalence has been continually declining from 9.6% in the year 2001 to 5.6% in 2011 [16]. However, in the subsequent surveys including this one, the prevalence has been inclining. In the 2017 HSS, HIV prevalence was 6.1%, and it is 6.3% in the current HSS. The incline in prevalence after 2011 may indicate ART-associated improved survival as a result of the introduction of Option B+ in Tanzania in September 2013. Option B+ recommended initiating ART to all pregnant women living with HIV regardless of their CD4 count [15, 16, 27]. Option B+ is also an intervention behind the global reduction of MTCT and indeed this has also resulted in increased fertility among women living with HIV [11, 13, 28, 29]. Improved survival and fertility among women living with HIV may also be evident in this survey as 70% of pregnant women living with HIV initiating ANC knew their HIV-positive status.

It is critical to realize that the increasing prevalence is also driven in part by the new infections. Tanzania is among the countries with the highest HIV incidences worldwide, most of which occur among females 15–24 years old [7]. As seen in this HSS, although pregnant women 15–24 years had the lowest prevalence their HIV status awareness was also lowest and we speculate the majority may be new HIV infections. Trends of HIV prevalence among 15–24 years old from several sentinel surveillances has been used as a proxy to monitor HIV incidence [30, 31]. In Tanzania, HIV incidence has been declining and similarly HIV prevalence among pregnant women 15–24 years has been continually declining from 7.6% in 2001 to 3% in this survey [16]. However, it is worth noting that the changes in the survival of PLHIV may have affected the reliability of the proxy [31]. An updated assessment of the prevalence of HIV among young women as a proxy of incidence is needed to ensure its applicability in the current era.

HSS has also been used to inform on the prevalence and trend of HIV infections in the general population [14]. In this survey, the prevalence of HIV infection among pregnant women in Tanzania mainland was 5.9%. It was higher compared to that of the general population (4.7%), but lower than that of women (15–49 years) in the general population (6.4%) as reported in the population-based survey in 2017 [11]. HSS normally would overestimate the prevalence in the general population and among women of reproductive age, especially adolescents because of differences in the levels of sexual activity [18]. Indeed, the prevalence of HIV infection among pregnant women aged 15–49 in Tanzania mainland from 2003/04 and 2006/07 HSS were higher than those of women from the parallel population-based surveys [15, 27, 32–34]. Conversely, during 2011, 2017, and the current HSS, the HIV estimates among pregnant women were lower than that of women in the parallel population surveys [11, 16, 27, 32]. The lower prevalence in the recent HSS than in the general population surveys may also reflect ART-associated survival of women living with HIV. As a result, HIV infections are more pronounced in the population-based surveys than in the surveillance of pregnant, because HIV infection has shifted toward older, less-fertile women [29, 35]. Although fertility among women living with HIV has improved in Tanzania and other similar settings, it is still lower than that of women not living with HIV, especially among older ones [36–38]. Therefore, estimating HIV prevalence in the general population using the HSS findings should account for this shift in HIV and fertility patterns [29, 35].

Similar to the previous ANC surveys and other population-based surveys, the 2020 ANC HSS observed wide regional variations in HIV prevalence ranging from 1.9% in the Manyara region to 16.4% in the Njombe region. Iringa region (13.6%) and Mbeya region (11.9%) ranked second and third for HIV prevalence, in line with findings from the previous surveys [27]. The persistently high burden in these regions could partly be explained by the continued efforts by the Government of Tanzania and its stakeholders to address AIDS-related deaths resulting in improved survival for people living with HIV infection. However, the persistently high prevalence and low HIV status awareness in these regions may indicate the potential for a high transmission rate. Consequently, the high prevalence, low HIV status awareness, and subsequently low ART coverage may further fuel onward transmission and increase the incidence of HIV infections [39].

The overall HIV status awareness has increased from 43% in 2017 to 70% in 2020, but it is still below the 95% target. Only one region (Ruvuma) reached 90% in this survey. Although 91.6% of pregnant women who were aware of their HIV-positive status were on ART, overall, only 66.6% of all women living with HIV in this survey were diagnosed and on ART. It is worth stressing that the low level of HIV status awareness and ART coverage persisted even in sensitivity analyses. Closing this gap in HIV testing and treatment is a critical part of the response to the HIV epidemic as it has significant implications for preventing onward transmission of HIV and its impact on the incidence at the population level [40]. Tanzania, which is also among the countries with the highest HIV incidences globally, urgently needs to strengthen the existing HIV testing services (HTS) and employ new and innovative ways to get people tested for HIV. As revealed in this study, the expansion of HTS also needs to be done strategically by prioritizing regions and population groups with lower coverage of HTS and ART, such as young women and adolescents. Coverage was almost twice for non-primigravida compared to primigravida women indicating that integration of HTS into other services increases the uptake of HTS which is in line with the findings and recommendations of other studies [41–43]. These should go hand in hand with linkage to treatment and care [3]. Efforts should be directed toward the most vulnerable areas with high prevalence, low HIV status awareness, and low ART coverage [44]. It is relatively more challenging, logistically and financially, to determine incidences, but data from surveillances such as this one may help inform on the incidence and implement interventions. Additionally, through surveys such as this, a score for predicting incidence using the data from surveys may be developed and validated.

The analysis of factors associated with HIV determined that older age and lower level of education were associated with higher HIV prevalence. The age factor reflects the increased survival of PLHIV in the era of ART. Since this was a cross-sectional survey, it is not surprising that older pregnant women were more likely to be found with HIV infection than their younger counterparts. A cross-sectional survey, by nature of its design, studies survivors of a chronic infection like HIV and hence high prevalence among them. The prevalence of HIV decreased with an increase in education level like in the population-based HIV survey in Tanzania [11]. Education attainment delays sexual debut and keeps girls in school which offers a protective environment against unwanted pregnancies and risky sexual behaviors [45]. Evidence in the current survey is contrary to the previous surveys, where the prevalence of HIV was higher among people with higher levels of education [15]. This was thought to be attributed to a lack of access to HIV preventive methods and their positions in society rendered them to risky sexual behaviors [15].

In the current survey, pregnant women in the marital union had the lowest prevalence of HIV infection (5.7%) compared to those outside of the marital union. The highest prevalence was observed among pregnant women reported to be widowed (23.0%), divorced or separated (22.4%), and those who were single (10.4%). The relationship between marital status and HIV infection is complex and largely understudied but cultures and sexual behaviors have a large role in this interplay [46, 47]. Consistent with our findings, the recent population demographic survey indicated a lower lifetime number of sexual partners among married women compared to women outside the marital union. Married women are also more likely to use condoms during sexual intercourse with a person who is not their husband or living with them compared to unmarried women [48], preventing them from new HIV infections.

Living in urban and semi-urban areas was associated with higher HIV infections compared to living in rural areas in this survey. A similar pattern has been observed among pregnant women over the years in Tanzania [27]. The sexual behavioral differences between women in urban and rural may influence this observation. Women in rural areas have a lower average number of lifetime sexual partners and fewer have sexual intercourse outside of marital union or with a non-cohabiting partner compared to women in urban areas [48]. However, the proportion of condom use in these instances is comparable in the two settings [48].

It has been observed in several studies that individuals who are affected with HIV have lower participation in the labor force particularly in manual labor and understandably, the severity of the disease is associated with lower employment [49, 50]. Nevertheless, in this nationwide survey, the prevalence of HIV was higher among those who reported being employed or self-employed. This observation may be a result of the survival and stability of those who are employed compared to those who are not employed. People affected with HIV who are employed are likely to test for HIV, be diagnosed earlier, and initiate ARTs with better adherence than people who are not employed [49, 51, 52]. This can in turn translate to better survival and stable health among those employed compared to those who are not employed [51, 52]. Employment increases financial and economic empowerment among women, which may increase access to healthcare services, reduce HIV-related morbidity and mortality, and improve the quality of life for PLHIV [51, 53].

This study is not without some limitations. Most of the sites used in this survey were inherited from previous surveys and there are some demographic changes of the sites over time and newer PMTCT sites which could have featured in the sampling frame had sampling been conducted again. Thus, the choice to inherit previous PMTCT sites might have narrowed our potency to be as representative as possible and the weights used were regional weights and due to the lack of updated and reliable strata specific data for each region. In addition, the distribution of characteristics of non-responders were not collected which could have influenced the results. However, the non-response rate was exceedingly low (<2%), and this survey had substantial sample size of over thirty-eight thousand pregnant women on their first ANC visit.

## Conclusion

One in every twenty pregnant women attending ANC in Tanzania mainland is living with HIV. Prevalence varies between regions, but HIV is especially prevalent among older pregnant women, those with lower or no formal education, those outside marital unions, and those living in urban and semi-urban areas. Only seven out of ten pregnant women living with HIV are aware of their HIV-positive status. However, ART is covered in about nine out of ten pregnant women living with HIV who were aware of their HIV-positive status. Behind the global fast-target to end HIV/ AIDS, about a third of pregnant women living with HIV initiating ANC were not on ART. Interventions to increase HIV testing and linkage to care among women of reproductive age should be intensified.

## Supporting information

S1 Checklist STROBE statement—a checklist of items that should be included in reports of observational studies. (DOCX)
